# S/Se-Terchalcogenophene-C_60_ Dyads: Synthesis and Characterization of Optical and Photosensitizing Properties

**DOI:** 10.3390/ma16072605

**Published:** 2023-03-24

**Authors:** Radosław Motyka, Klaudia Nastula, Piotr Pander, Damian Honisz, Mateusz Tomczyk, Karol Erfurt, Agata Blacha-Grzechnik

**Affiliations:** 1 Faculty of Chemistry, Silesian University of Technology, Strzody 9, 44-100 Gliwice, Poland; 2 Centre of Polymer and Carbon Materials, Polish Academy of Sciences, Curie-Sklodowskiej 34, 41-819 Zabrze, Poland; 3 Centre for Organic and Nanohybrid Electronics, Silesian University of Technology, Konarskiego 22B, 44-100 Gliwice, Poland; 4 Department of Physics, Durham University, South Road, Durham DH1 3LE, UK

**Keywords:** fullerene dyads, photosensitization, singlet oxygen, reactive oxygen species

## Abstract

Fullerenes have been long investigated for application as singlet oxygen sources. Even though they possess high photosensitizing efficiency, their practical use is still limited, mostly because of insufficient absorption of visible and/or near-infrared light. This limitation can be overcome by introducing organic chromophores that absorb longer-wavelength light, either by covalent attachment to C_60_ or by its encapsulation in a polymeric matrix. In this work, we investigated the photosensitizing properties of the C_60_ molecule functionalized with organic units comprising thiophene or selenophene rings. The chemical structures of the synthesized dyads were characterized by nuclear magnetic resonance (NMR) spectroscopy and mass spectrometry. The influence of the S/Se atoms and vinyl linkage between the organic unit and C_60_ on the absorptive and emissive properties of the dyads was investigated and correlated with their photosensitizing activity. For the latter, we used a standard chemical singlet oxygen trap. A selected dyad C_60_ThSe_2_ was also applied as a source of singlet oxygen in a model photocatalyzed synthesis of the fine chemical juglone from 1,5-dihydroxynapthalene.

## 1. Introduction

Singlet oxygen (^1^O_2_) plays an important role in many biological and chemical processes owing to its highly oxidative properties and high reactivity. It was first observed in the 1960s and remains within the area of interest of scientists as a useful reagent in oxidation processes, in fine chemical synthesis, or in medicine, e.g., photodynamic therapy (PDT) [1,2,3,4]. ^1^O_2_, a form of reactive oxygen species (ROS), can be formed in various (photo)chemical and physical processes. Among these processes, photosensitization is considered the most favorable example. This process relies on the presence of three components: (1) a photoactive molecule, i.e., a photosensitizer (PS); (2) molecular oxygen; and (3) light of a suitable wavelength to be absorbed by the PS. In simple terms, the light-activated production of ^1^O_2_ undergoes three major steps: (i) absorption of light by the PS to form an excited singlet state (^1^PS*); (ii) formation of a triplet excited state of the PS (^3^PS*) by intersystem crossing (ISC); and (iii) Dexter electron transfer from the ^3^PS* to triplet oxygen, yielding singlet oxygen [4]. Even though several groups of organic photosensitizers have been investigated, such as porphyrins, phthalocyanines, and other transition metal complexes [4], the search for the ideal PS molecule with high photostability and optimal optical and photosensitizing properties is ongoing. 

Fullerene (C_60_) has attracted significant attention in the last years due to its good electron accepting/transporting properties, leading to its broad use in optoelectronic devices [5,6] or as a singlet oxygen source in photochemical processes [7,8,9,10]. Unsubstituted fullerene produces singlet oxygen with very high efficiency [11,12,13] but suffers from low absorption in the visible region of the electromagnetic spectrum and low solubility in organic solvents (while being insoluble in water [14]), which strongly hinders its practical use. It has been shown that both problems can be solved by appropriate functionalization of the fullerene unit [11,15,16,17,18,19]. A number of works have shown an enhancement in the optical and photosensitizing properties of fullerene-based photosensitizers. For example, several fullerene dyads, triads, and tetrads with visible to near-infrared light-absorbing groups have been reported [20,21,22,23,24,25,26,27]. However, due to the low solubility of C_60_, its functionalization is usually arduous, especially when introducing functional groups, which may lead to even lower solubility. 

In our previous works on C_60_ dyads, we have shown that fullerene having only thiophene or selenophene substituents can be deposited as a layer in an electrochemical polymerization and can be effectively used as a heterogeneous source of singlet oxygen [28,29]. In this work, we aimed to investigate the optical and photosensitizing properties of those *simple* fullerene dyads, with three units of five-membered heterocycles serving as light-absorbing antennas. In contrast to our previous works dealing with the solid photoactive systems, we characterized the photoactivity of the C_60_ dyads in solution phase, investigating the effect of a heavier heterocyclic chalcogen and the presence of a vinyl spacer between the C_60_ unit and the antenna. Thus, three fullerenes with thiophene/selenophene side substituents were synthesized, and their structures were confirmed by means of nuclear magnetic resonance (NMR) spectroscopy and mass spectrometry. The optical properties were investigated in solution with UV–Vis absorption and fluorescence spectroscopy. The photosensitizing properties were investigated using an indirect method with a tetraphenylcyclopentadienone (TPCPD) ^1^O_2_ trap. The oxidation of 1,5-dihydroxynapthalene to juglone was used as a model homogenous photo-oxidation process to demonstrate the effectiveness of our dyads in generating singlet oxygen. Finally, we discussed the influence of the dyad structure on its optical and photosensitizing properties, demonstrating the significance of the ISC occurring directly on the antenna.

## 2. Materials and Methods

### 2.1. Materials

**Figure 1:**
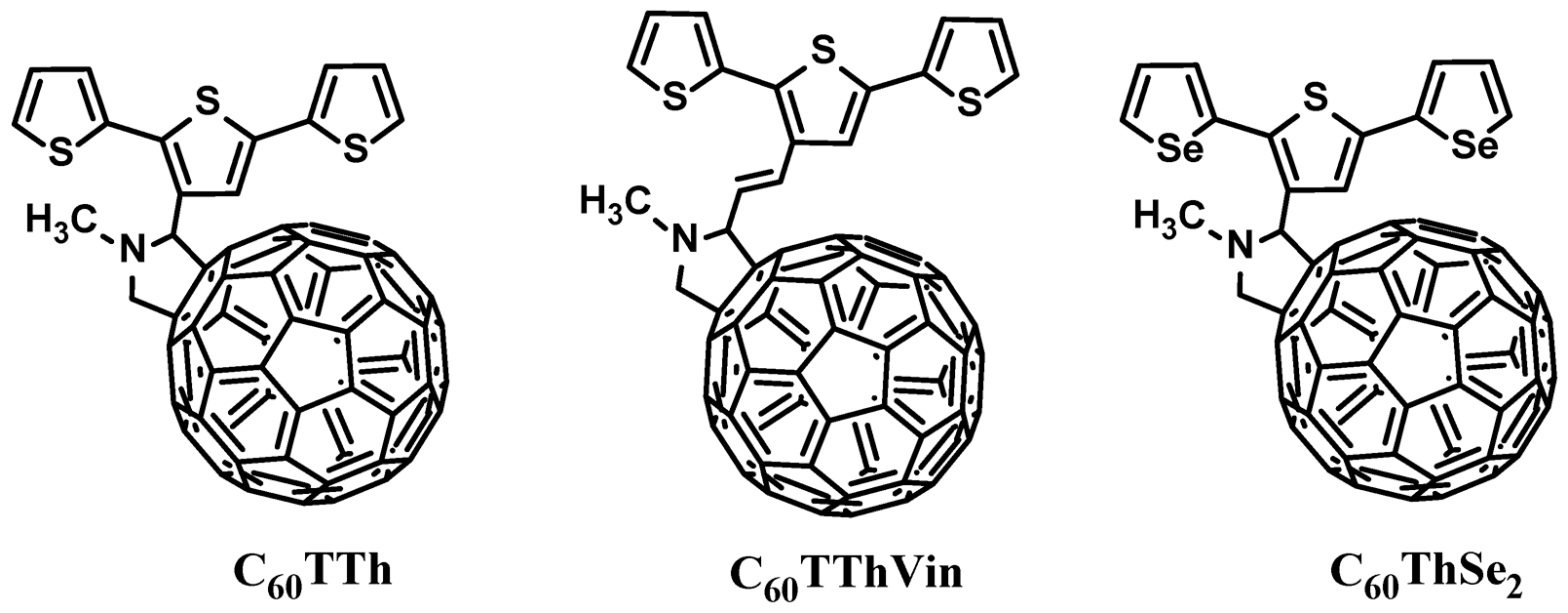
Investigated C_60_ dyads.

Thiophene– and selenophene–C_60_ dyads (Figure 1) were synthesized based on previously reported procedures [30,31,32,33]. The detailed synthetic route and the identification of the obtained products are shown in the Supporting Information. 2,2′:5′,2″-Terthiophene (TTh) and fullerene C_60_ were purchased from Alfa Aesar, Haverhill, MA, USA (purity 99%) and Sigma Aldrich, St. Louis, MO, USA (purity 98%), respectively. Dichloromethane (HPLC grade, Sigma Aldrich, St. Louis, MO, USA) was used as a solvent for UV–Vis spectroscopy and photophysical studies. Tetraphenylcyclopentadienone (TPCPD) (Acros Organics, Geel, Belgium, purity 99%) in dichloromethane was used as a specific ^1^O_2_ quencher. Perinaphthenone (Sigma Aldrich, St. Louis, MO, USA, purity 97%) was applied as a reference for the determination of the quantum yield of singlet oxygen production. Photooxidation of 1,5-dihydroxynaphtalene (DHN, Sigma Aldrich, St. Louis, MO, USA, purity 97%) was conducted in dichloromethane: methanol (Across Organics, Geel, Belgium, purity 99.9%) mixture (9:1 *v*/*v*).

### 2.2. Characterization of Optical and Photosensitizing Properties of Thiophene/Selenophene Dyads

UV–Vis spectra of fullerene dyads in dichloromethane were collected in a 2 mm quartz cuvette (Hellma Analytics, Müllheim, Germany) with a Hewlett Packard 8452A UV–Vis spectrometer. The concentration of dyads varied between 6.25•10^−3^ and 0.1 mM. 

Photoluminescence (PL) spectra in solutions were recorded using a QePro compact spectrometer (Ocean Optics). Excitation curves were recorded using a Horiba Fluorolog fluorescence spectrometer. Time-resolved photoluminescence measurements in solution were recorded with a Horiba DeltaFlex TCSPC system using a 405 nm Delta Diode light source.

The photosensitizing properties of thiophene/selenophene dyads were investigated with an indirect method using UV-Vis spectroscopy with a 0.05 mM solution of tetraphenylcyclopentadienone (TPCPD) in dichloromethane [34] as a specific singlet oxygen trap. The concentration of photosensitizer was 0.025 mM. The progress of the reaction between TPCPD and ^1^O_2_ was monitored with UV-Vis spectrophotometry using the 510 nm absorption band of TPCPD as a reference. We used standard 10 mm × 4 mm quartz cuvettes (Hellma Analytics), and the experimental setup was arranged in such a way that the irradiation beam with a ca. 0.5 cm^2^ cross-section was perpendicular to the optical path of the UV-Vis spectrophotometer. 365 nm fiber-coupled LED (Thorlabs, Bergkirchen, Germany) or xenon lamp (Thorlabs) served as the excitation light source in the experiment. The UV-Vis spectra were acquired at time intervals in which clear changes in the absorbance of the chemical trap could be observed. 

The quantum yield of singlet oxygen generation was estimated with a relative method using Equation (1), with perinaphthenone (PN) as a reference (*Φ*_PN_ = 0.95 in dichloromethane) [35].

(1) $$\Phi_{i}=\Phi_{\mathrm{PN}}\cdot \frac{m_{i}}{m_{\mathrm{PN}}}\cdot \frac{\alpha_{\mathrm{PN}}}{\alpha_{i}}$$

where the indices i and PN indicate fullerene dyad and perinaphthenone, respectively; *Φ* is the quantum yield of singlet oxygen photosensitization; *m* is the slope of temporal change of absorbance at 510 nm of TPCPD; *α* is given by α = 1 − 10^–A^, where A is absorbance at 365 nm [36,37,38].

The selected fullerene dyads and unsubstituted C_60_ were used as sources of ^1^O_2_ in a model photooxidation reaction applicable for fine chemical synthesis, i.e., the oxidation of DHN. Firstly, in situ measurements were undertaken using the measurement setup described above with a 1 mM solution of DHN in CH_2_Cl_2_:CH_3_OH. The progress of the reaction was monitored by observing an increase in absorbance at 420 nm. Photo-oxidation of DHN was also performed on a laboratory scale using a photoreactor (100 mL of borosilicate glass flask) and a xenon lamp (Thorlabs) as a light source. All experiments were conducted on the freshly prepared solution of fullerene dyads.

## 3. Results and Discussion

### 3.1. Synthesis

**Scheme 1:**
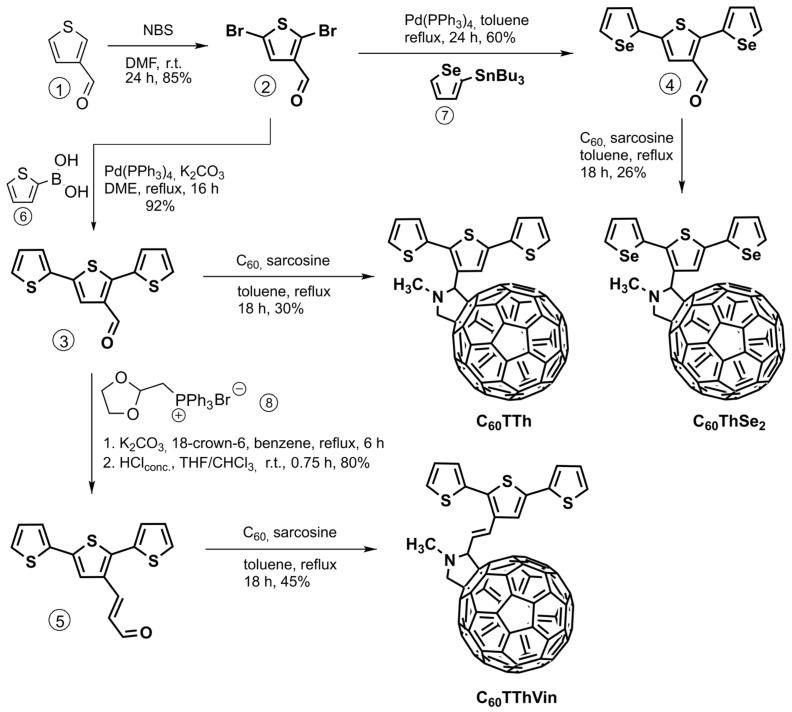
Synthetic path for investigated C_60_ dyads.

All compounds were synthetized using 3-formylthiophene (**1**) as a starting material. The synthetic path is shown in Scheme 1.

In the first step, thiophene aldehyde (**1**) was brominated with the use of N-bromosuccynimide [30] to give product **2,** which was coupled with 2-thienylboronic acid (**6**) in the Suzuki–Miyaura reaction [31] and with 2-tributylstannylselenophene (**7**) using the Stille reaction [32], which led to 2,2′:5′,2″-terthiophene-3′-carbaldehyde (**3**) and 2,5-di(selenophen-2-yl)thiophene-3-carbaldehyde (**4**). Compounds **3** and **4** were used to obtain the final dyads C_60_TTh and C_60_ThSe_2_ by reacting them with buckminsterfullerene and sarcosine (N-methylglycine) in the 2 + 3 dipolar cycloaddition (Prato reaction) [32]. C_60_TThVin was synthetized in a similar manner, but in the last step (Prato reaction), 3-[(2,2′:5′,2″-terthiophen)-3′-yl]acrylaldehyde (**5**) was used. Compound **5** was prepared from derivative **3** by the Wittig reaction with (1,3-dioxolan-2-ylmethyl)triphenylphosphonium bromide [33] and subsequent hydrolysis of the acetal group.

### 3.2. Characterization of Optical and Photosensitizing Properties of Thiophene/Selenophene Dyads

#### 3.2.1. UV–Vis Absorption and Fluorescence Spectroscopy

**Figure 2:**
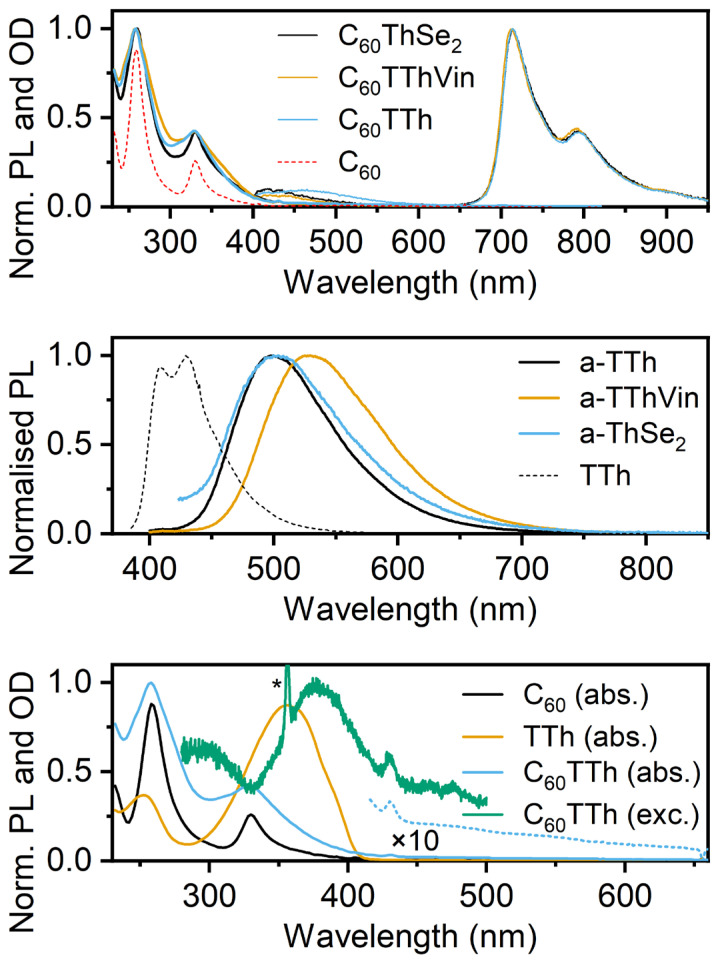
(**top**) Normalized UV–Vis absorption and photoluminescence (PL) spectra (c = 5 × 10^−5^ M) of dyads. Absorption spectrum of C_60_ is shown for reference. (**middle**) PL spectra of **3** (a-TTh), **4** (a-ThSe_2_), **5** (a-TThVin), and TTh (c = 10^−5^ M). (**bottom**) Absorption spectra of C_60_, TTh (terthiophene), and **C_60_TTh** together with the excitation spectrum of **C_60_TTh** (λ_col_ = 714 nm). Asterisk (*) indicates harmonic peak of the collection wavelength. All experiments were recorded in CH_2_Cl_2_.

Figure 2 (**top**) presents the normalized UV–Vis and PL spectra of the C_60_ dyads in CH_2_Cl_2_, and absorption spectra of the model C_60_ and TTh units. A summary of the photophysical properties of the dyads is presented in Table 1. The main absorption bands of the nonfunctionalized fullerene and terchalcogenophene-decorated C_60_ units are located within the 250–400 nm region and display a clear overlap. Weak absorption originating from the C_60_ extends out from 400 nm onward and beyond 600 nm. The absorption spectra of dyads are likely a superposition of the absorption originating from the two constituent units, with distinctive maxima at ~260 nm and ~330 nm originating from the C_60_ unit [11]. As the sharp absorption maxima characteristic for the C_60_ unit are retained in the decorated C_60_; therefore, it is likely the electronic structure of the fullerene unit is only weakly affected by functionalization. Therefore, the two constituent components of the dyads are electronically decoupled, hence behaving as isolated π systems. When excited with λ_exc_ = 365 nm, the dyads display weak photoluminescence characteristic of the C_60_ unit, with a maximum at 714 nm and a vibronic progression of ~1400 cm^−1^. The weak PL of the C_60_ dyads likely originates from the low luminescence yield of the C_60_ unit [39]. The weak PL in the ~400–500 nm region can be ascribed to the residual fluorescence of the antennas.

Precursors **3**, **4**, and **5**, although structurally close to the respective dyad antennas, are suboptimal models for the systems, as demonstrated by the difference in PL spectra between **3** (a-TTh) and TTh (Figure 2 (**middle**)). However, they are still useful for explaining certain features of the luminescent behavior of the respective dyads. We note that the presence of a Se atom instead of a S atom has a minor effect on their photoluminescence spectrum, leading to a red shift by a few nanometers. On the contrary, the vinyl linker appears to have a profound effect on the PL spectra, which can be connected to extended conjugation by direct linkage to the central Th unit. The photoluminescence lifetime of the antennas is strongly linked to their structure—we observed the longest decay lifetime for **3** (τ = 1.8 ns) and a shorter lifetime of the analogue with a vinyl linker **5** (τ = 0.3 ns). The shortest lifetime is observed for **4** (τ = 0.1 ns) (Figure S1), which includes heavy Se atoms replacing some of the sulfur in the chalcogenophene rings. The direct reasons for the analogues with the vinyl linker showing shorter PL lifetimes than the nonvinyl dyads are unclear; however, the behavior of **4** is consistent with the heavy atom effect. In this case, the heavy Se atom induces faster rates of intersystem crossing, leading to fluorescence quenching and higher triplet populations originating from optically excited species.

**Figure 3:**
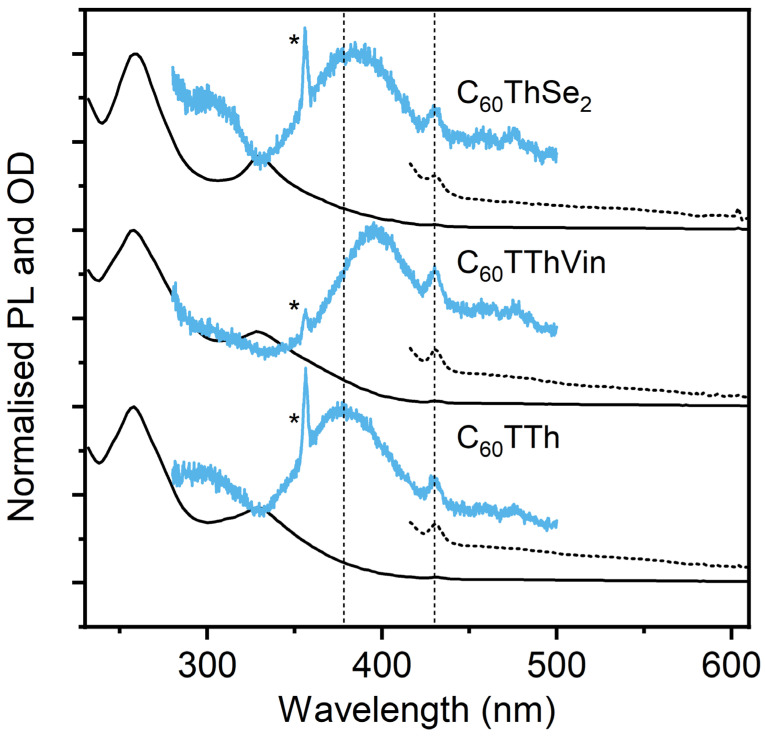
Absorption (black continuous lines) and excitation (λ_col_ = 714 nm, cyan continuous lines) spectra of the C_60_ dyads. Dashed curves indicate fragments of absorption spectra multiplied by a factor of 10. Vertical dashed lines are shown as an eye guide. Asterisk (*) indicates harmonic peak of the collection wavelength. All experiments were recorded in CH_2_Cl_2_.

A further investigation of the **C_60_TTh** dyad (Figure 2 (**bottom**)) confirms the tentative hypothesis of the role of the TTh antenna in the system. The excitation curve recorded with collection at 714 nm resembles the shape of the TTh absorption and does not display the C_60_ absorption peak at ~330 nm. This is crucial evidence for the role of the antenna in the absorption of light in the 300–400 nm region. Interestingly, the excitation curve displays a sharp maximum at ~430 nm, which can also be observed in the absorption spectra of **C_60_TTh**. C_60_ displays a similar sharp absorption peak at ~405 nm; therefore, we ascribe the ~430 nm band to the C_60_ unit—a redshift of this band with respect to C_60_ can be rationalized by a change in electronic structure resulting from functionalization. Hence, the C_60_ unit significantly contributes to the excitation curve from ~420 nm on. We observed similar behavior in the other dyads (Figure 3), with the ~430 nm C_60_ absorption band retained in all cases, confirming its origin from said unit. The maxima of the excitation curves display small shifts that correspond to the changes in the PL spectra of the respective model antennas in Figure 2 (**middle**), hence confirming their antenna-based origin.

#### 3.2.2. Photosensitizing Properties of Dyads

From a wide range of possible singlet-oxygen-specific traps, we selected tetraphenylcyclopentadienone as the model quencher. This molecule is stable under white light illumination, can be used in dichloromethane, and—importantly—is selective toward ^1^O_2_ [34].

**Figure 4:**
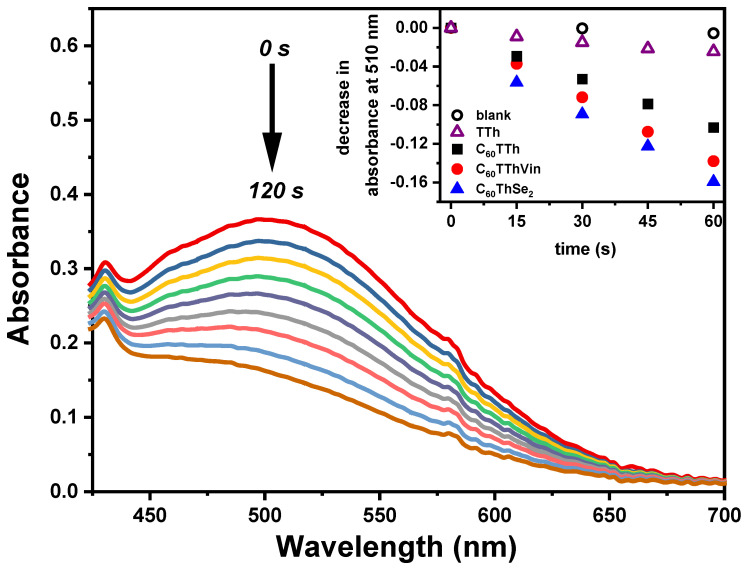
Set of UV–Vis spectra of tetraphenylcyclopentadienone (TPCPD) in CH_2_Cl_2_ in the presence of **C_60_TTh** upon illumination with xenon lamp. *Inset*: temporal decrease in the absorbance of tetraphenylcyclopentadienone (TPCPD) at 510 nm in the absence of PS (blank), the presence of TTh, **C_60_TTh**, **C_60_TThVin**, and **C_60_ThSe_2_** during illumination with the xenon lamp.

Figure 4 presents the UV–Vis spectra of TPCPD in CH_2_Cl_2_ recorded during the irradiation of **C_60_TTh**. A gradual decrease in the absorption band intensity at 510 nm indicates the reaction of TPCPD with singlet oxygen produced by the fullerene dyad **C_60_ThSe_2_**. Similar results were obtained with the other investigated dyads. The comparison (Figure 4 inset) shows that no drop in the TPCPD absorption band was recorded without the photosensitizer being present in the solution (blank), which demonstrates the stability of the TPCPD under illumination. The highest yield of TPCPD oxidation after 60 s was observed for **C_60_ThSe_2_**, while the lowest was observed for **C_60_TTh**. As reported earlier [40], TTh shows photosensitizing properties (Figure 4 inset); however, we observed a very low efficiency of singlet oxygen generation. As shown in Table 1, the introduction of a vinyl linker or replacement of two thiophene units with selenophene leads to an increase in the quantum efficiency of the photosensitization process. 

**Scheme 2:**
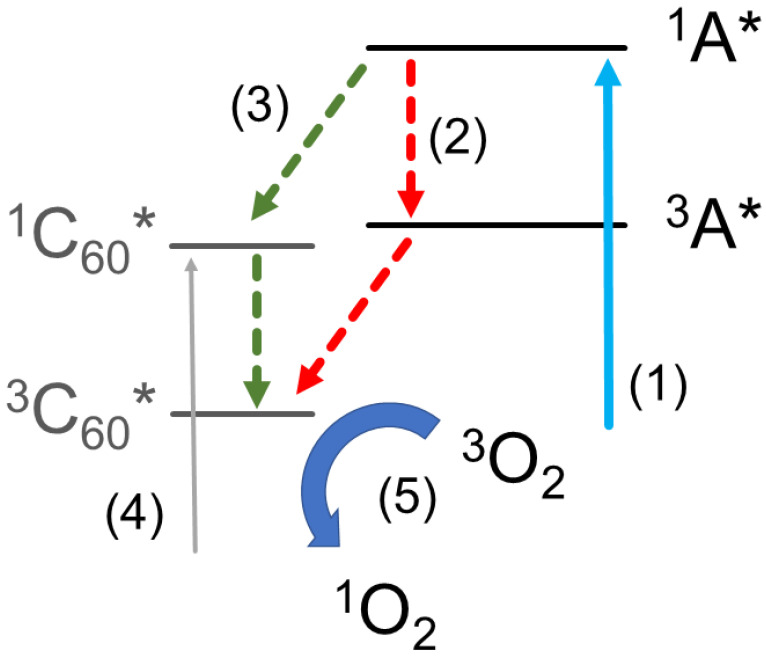
Schematic representation of the proposed mechanism for singlet oxygen generation by the C_60_ dyads: (1) absorption of light by antenna A; (2) intersystem crossing within the antenna, ^1^A*→^3^A*, followed by triplet energy transfer from the antenna A to the C_60_, ^3^A*→^3^C_60_* (red trace); (3) singlet energy transfer from antenna A to C_60_, ^1^A*→^1^C_60_*, followed by intersystem crossing within C_60_, ^1^C_60_*→^3^C_60_* (green trace); (4) direct absorption of light by C_60_ leading to ^1^C_60_* states; (5) interaction of triplet oxygen ^3^O_2_ with ^3^C_60_* to form singlet oxygen ^1^O_2_.

The mechanism for singlet oxygen (^1^O_2_) generation by fullerene dyads suggested in the literature involves energy transfer from the photoexcited singlet states of the light-absorbing antennas (^1^A*) to the singlet state of the fullerene (^1^C_60_*), followed by intersystem crossing (ISC) to the triplet state of the C_60_ unit (^3^C_60_*), which is the final state responsible for generating ^1^O_2_ [11,20,41,42,43,44]. However, our results point at this mechanism not being the sole mode in which ^1^O_2_ is generated by our systems (Scheme 2). We noted that higher rates of intersystem crossing in the Se-containing dyads correlated with the largest *Φ*_Δ_ values, which are inherently related to the final triplet populations localized on the C_60_ units (^3^C_60_*). This behavior suggests that the supportive mode for triplet generation in our systems is intersystem crossing from the ^1^A* to the ^3^A*, followed by triplet energy transfer to the ^3^C_60_*. Importantly, the C_60_ unit may also absorb light directly and produce ^1^O_2_ without the intermediary role of the antenna. The aforementioned mechanism suggested in the literature is still at play in our systems, as clearly demonstrated by the quenching of fluorescence from antennas by the C_60_ unit in Figure 2. We think that this proposed mechanism involving two distinct ISC pathways supplements those presented earlier in the literature. It is possible owing to the relatively fast ISC rates of selenophenes used as antennas. The popular BODIPY units used in related systems often display negligible ISC rates on their own, thus pointing at the ISC necessarily occurring outside of the BODIPY antenna unit and on the C_60_ moiety.

#### 3.2.3. DHN Photooxidation

The oxidation of 1,5-dihydroxynaphtalene (DHN) to juglone (5-hydroxy-1,4-naphthoquinone), an anthelmintic drug, is classified as a fine chemical process that naturally occurs in plants, especially in black walnut [45,46]. We used this reaction as a model to demonstrate the photosensitizing properties of our fullerene dyads. 

**Figure 5:**
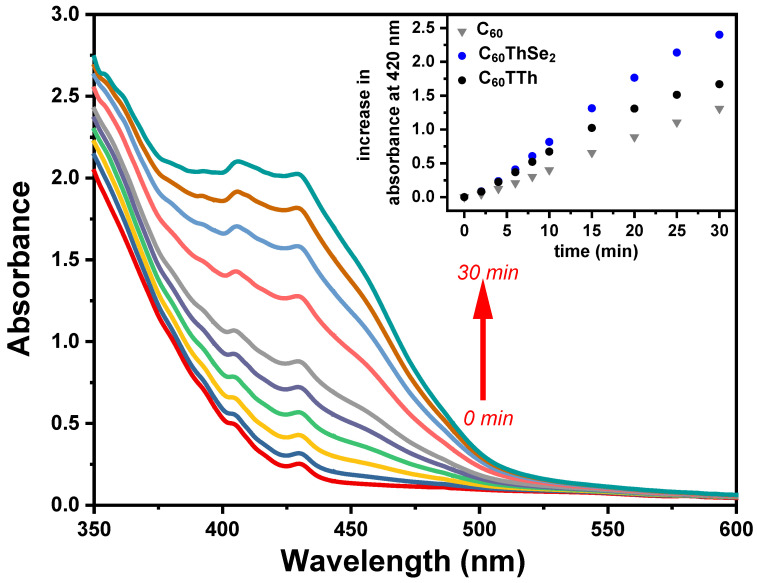
Set of UV–Vis spectra of DHN in CH_2_Cl_2_ recorded in the presence of **C_60_ThSe_2_** during illumination with xenon lamp. *Inset:* Increase in the absorbance at 420 nm over time recorded in the presence of C_60_, **C_60_TTh**, and **C_60_ThSe_2_** during illumination with xenon lamp.

At first, we investigated the oxidation process in situ using the C_60_ dyads as a source of singlet oxygen and a xenon lamp as a light source. The progress of DHN oxidation can be monitored either as a decrease in the absorbance of DHN at 298 nm or as an increase in the absorbance of the absorption band of juglone at 420 nm [47,48]. Because the investigated dyads strongly absorb at ca. 300 nm, we chose the latter option as more reliable. Figure 4 presents a set of UV–Vis spectra of a DHN solution containing **C_60_ThSe_2_** collected during irradiation. We observed that the absorbance of juglone at 420 nm gradually increased, indicating oxidation of DHN with singlet oxygen produced by the fullerene dyad. The comparison of **C_60_ThSe_2_** with **C_60_TTh** and C_60_ (Figure 5 inset) indicated that the selenophene-containing dyad shows the highest efficiency of singlet oxygen generation, nearly two-fold higher than for the unsubstituted C_60_. The lower reaction yield with **C_60_TTh** than **C_60_ThSe_2_** is in agreement with results obtained using TPCPD described above. This is mainly due to the higher *Φ*_Δ_ owing to the heavy atom effect but also a slightly higher absorbance of **C_60_ThSe_2_**.

Finally, we used **C_60_ThSe_2_** as the most effective singlet oxygen-generating agent for the synthesis of juglone on a preparative scale. For this purpose, 50 mL of a 25 mmol solution of DHN in a mixture of CH_2_Cl_2_:CH_3_OH (9:1 *v*/*v*) was placed in a 100 mL round-bottom borosilicate glass flask and was irradiated with a xenon lamp in the presence of 1% (by mass) of the photosensitizer and a constant flow of oxygen through the reaction mixture. The progress of the reaction was monitored by thin-layer chromatography using CH_2_Cl_2_ as the mobile phase and commercial juglone as a reference. The reaction was terminated after five hours when no increase in the amount of the target product was observed on the TLC plates. The product was isolated from the reaction mixture by column chromatography to give pure juglone (NMR spectra consistent with the commercial product) in a 40% yield. Although the yield achieved in this experiment was modest, it serves as a clear proof of concept for the use of our dyads for synthesis on a scale larger than micrograms. 

## 4. Conclusions

In summary, fullerene dyads with organic units comprising three chalcogenophene rings were synthesized and their optical and photosensitizing properties characterized. It was shown that decoration with thiophene and selenophene units leads to a significant improvement in effective light absorption in the 300–420 nm region of the electromagnetic spectrum. All of the investigated C_60_ dyads exhibited photosensitizing properties, with the quantum efficiency of singlet oxygen generation being the highest for the selenium-containing dyad. They displayed near-infrared luminescence at 714 nm, characteristic of the C_60_ unit, indicating efficient energy transfer from the antenna to the fullerene despite the formal electronic decoupling of the two units. We suggest a two-pathway mechanism for the generation of triplet states at the C_60_ units: (1) *singlet* energy transfer from the antenna to the fullerene, with the ISC occurring on the latter; (2) *triplet* energy transfer from the antenna to the fullerene. The effect of the mechanism (2) is evident in the prominent oxygen-generating properties of **C_60_ThSe_2_**.

It was shown that modification of the fullerene dyad with only three thiophene units can increase the yield of DHN oxidation by ca. 30% and the subsequent exchange of sulfur by selenium atoms by a further ca. 70% with respect to undecorated C_60_. Thus, the reported dyads can be considered a cost-effective alternative to the previously reported photosensitizers based on fullerene dyads.

## Figures and Tables

**Table 1:** Summary of absorption, emission and photosensitizing parameters of the investigated dyads.

Fullerene Dyad	λ_abs_/nm (ε/10^4^ M^−1^ cm^−1^)	λ_em_/nm	*Φ*_Δ_
**C_60_TTh**	258 (5.83), 328 (2.42), 430 (0.22)	714, 793, 900	0.22 ± 0.02
			
**C_60_TThVin**	258 (7.22), 328 (2.81), 430 (0.24)	714, 793, 900	0.26 ± 0.01
			
**C_60_ThSe_2_**	260 (9.02), 330 (3.29), 430 (0.23)	714, 793, 900	0.31 ± 0.02

## Data Availability

No data have been deposited.

## Supplementary Materials

The following supporting information can be downloaded at: https://www.mdpi.com/article/10.3390/ma16072605/s1, Synthesis and spectroscopic characterization of thiophene- and selenophene– C_60_ dyads. Figure S1. TCSPC traces in degassed CH_2_Cl_2_ solutions of 3, 4, and 5, c = 10^−5^ M. Collection wavelengths correspond to emission maxima in Figure 2 (middle).
