# Supplementary material for: S/Se-Terchalcogenophene-C60 Dyads: Synthesis and Characterization of Optical and Photosensitizing Properties
Source: Materials (Basel). 2023 Mar 24;16(7):2605. doi: 10.3390/ma16072605 (PMC10095422; doi:10.3390/ma16072605)
Supplement: Supplementary file 1 [file materials-16-02605-s001.zip › materials-2280392-supplementary.pdf]

materials

Supplementary materials

# S/Se-Terchalcogenophene-C60 Dyads: Synthesis and Characterization of Optical and Photosensitizing Properties

**Radosław Motyka [1,2,*], Klaudia Nastula [1], Piotr Pander [1,3,4], Damian Honisz [1], Mateusz Tomczyk [1], Karol Erfurt [1] and Agata Blacha-Grzechnik [1,3,*]**

[1] Faculty of Chemistry, Silesian University of Technology, Strzody 9, 44-100 Gliwice, Poland
[2] Centre of Polymer and Carbon Materials, Polish Academy of Sciences, Curie-Sklodowskiej 34, 41-819 Zabrze, Poland
[3] Centre for Organic and Nanohybrid Electronics, Silesian University of Technology, Konarskiego 22B, 44-100 Gliwice, Poland
[4] Department of Physics, Durham University, South Road, Durham DH1 3LE, UK
* Correspondence: radoslaw.motyka@gmail.com (R.M.); agata.blacha-grzechnik@polsl.pl (A.B.-G.)

## Synthetic Procedures

$^{1}$H- and $^{13}$C-NMR spectra were recorded on a Varian Unity Inova 300 MHz System (300 MHz for $^{1}$H and 75MHz for $^{13}$C), in specified solvent and with tetramethylsilane (TMS) as the internal reference. The chemical shifts (δ) are reported in parts per million (ppm) and the coupling constants (*J*) in Hertz. High resolution mass spectrometry analyses were performed on a Waters Xevo G2 Q-TOF mass spectrometer (Waters Corporation) equipped with an ESI source operating in positive- ion modes. Full-scan MS data were collected from 100 to 1000 Da in positive ion mode with scan time of 0.1 s. To ensure accurate mass measurements, data were collected in centroid mode and mass was corrected during acquisition using leucine enkephalin solution as an external reference (Lock-Spray TM ), which generated reference ion at m/z 556.2771 Da ([M+H]+) in positive ESI mode. The accurate mass and composition for the molecular ion adducts were calculated using the MassLynx software (Waters) incorporated with the instrument.
All anhydrous solvent were purchased from ACROS Organics and used as obtained. Dichloromethane (DCM for HPLC, 99.8%) – SigmaAldrich, Hexanes fraction from naphtha was obtained from Avantor Performance Materials Poland S.A.

**2,5-dibromo-3-formylthiophene** (**2**): In a 500 mL round-bottom flask 3-formylthiophene (**1**) (4.50 g, 40.0 mmol) was dissolved in 150 mL of anhydrous DMF and intensive stirring was begun. Then a solution of NBS (17.84 g, 100.0 mmol) in DMF (60 mL) was slowly added dropwise within 0.5 h. Reaction was carried out for 24 h at room temperature protecting the flask from sunlight. The mixture was poured into water (600 mL) and extracted with diethyl ether (3x75 mL). Combined organic phases were dried over anhydrous $MgSO_4$ and the solvent was removed by rotary evaporation to gain 9.63 g of crude product in the form of orange solid. The crude product was purified by column chromatography (silica gel, hexanes fraction from naphtha/dichloromethane mixture 2:1 v/v) to afford a slightly yellow solid of compound (**2**) (9.25 g, 85% yield).
$^{1}$H-NMR (300 MHz, $CDCl_3$) δ (ppm): 9.79 (s, 1H, CHO), 7.34 (s, 1H, $C^4$-H), $^{13}$C-NMR (75 MHz, $CDCl_3$) δ (ppm): 183.3 (CHO), 139.4, 128.8 ($C^4$-H), 124.3, 113.5.

**2,2':5',2''-terthiophene-3'-carbaldehyde** (**3**): In a 500 mL round-bottom flask 2,5-dibromo-3-formylthiophene (**2**) (8.00 g, 29.6 mmol), 2-thienylboronic acid (8.71 g, 68.1 mmol) and $Pd(PPh_3)_4$ (1.03 g, 0.89 mmol, 3% mol) were placed. Then the flask was purged with argon and degassed 1,2-dimethoxyethane (240 mL) followed by 1M solution of $Na_2CO_3$ in degassed water (150 mL) were added. Reaction mixture was intensively stirred and heated at reflux, under argon atmosphere for 16 h. After this time 1,2-dimethoxyethane was removed under diminished pressure and the residue was extracted with DCM. Combined extracts were dried over anhydrous $MgSO_4$ and DCM was

evaporated to give crude product in the form of brown oil, which was purified by column chromatography (silica gel, hexanes fraction from naphtha/dichloromethane mixture 1:1 v/v). Finally 7.45 g (92% yield) of product **3** was obtained in the form of yellow solid.
$^{1}$H-NMR (300 MHz, $CDCl_3$) δ (ppm): 10.08 (s, 1H, CHO), 7.56 (s, 1H, H-4′), 7.49 (dd, $^{3}J$ = 5.2 Hz, $^{4}J$ = 1.2 Hz, 1H), 7.31 (dd, , $^{3}J$ = 3.6 Hz, $^{4}J$ = 1.2 Hz, 1H), 7.29 (dd, $^{3}J$ = 5.2 Hz, $^{4}J$ = 1.2 Hz, 1H), 7.22 (dd, $^{3}J$ = 3.6 Hz, $^{4}J$ = 1.2 Hz, 1H), 7.16 (dd, $^{3}J$ = 5.2 Hz, $^{3}J$ = 3.6 Hz, 1H), 7.04 (dd, $^{3}J$ = 5.2 Hz, $^{3}J$ = 3.6 Hz, 1H). $^{13}$C-NMR (75 MHz, $CDCl_3$) δ (ppm): 185.2, 146.0, 137.9, 137.0, 135.7, 132.2, 129.4, 128.8, 128.4, 128.2, 126.0, 125.1, 122.6.

**2,5-di(selenophen-2-yl)thiophene-3-carbaldehyde** (**4**): In a 50 mL vacuum dried round-bottom flask 2,5-dibromo-3-formylthiophene (**2**) (1.61 g, 5.95 mmol, 1 eq.), 2-(tributylstannyl)selenophene (**3**) (5.00 g, 11.90 mmol, 2 eq.), $Pd(PPh_3)_4$ (0.34 g, 0.29 mmol, 0.05 eq.) and 25 mL of anhydrous, degassed toluene were placed. Reaction mixture was refluxed for 24 h under argon atmosphere. After this time solvent was evaporated and the residue was dissolved in dichloromethane (75 mL) to which saturated solution of potassium fluoride (75 mL) in water was added, obtained mixture was intensively stirred for 0.75 h and the phases were separated. Organic phase was washed with water, dried with anhydrous $MgSO_4$ and evaporated to dryness. Crude product was purified by column chromatography (silica gel, hexanes fraction from naphtha/dichloromethane mixture 1:2 v/v) to give 1.32 g (60% yield) of bright orange oil which solidified with time. $^{1}$H-NMR (300 MHz, $CDCl_3$) δ (ppm): 10.07 (s, 1H, CHO), 8.20 (dd, $^{3}J$ = 5.6 Hz, $^{4}J$ = 1.2 Hz, 1H), 7.96 (dd, $^{3}J$ = 5.6 Hz, $^{4}J$ = 1.2 Hz, 1H), 7.50 (s, 1H), 7.48 (dd, $^{3}J$ = 3.8 Hz, $^{4}J$ = 1.2 Hz, 1H), 7.43 – 7.35 (m, 2H), 7.27 (dd, $^{3}J$ = 5.6, $^{3}J$ = 3.8 Hz, 1H).

**(*E*)-3-(2,2':5',2''-terthiophen-3'-yl)prop-2-enal** (**5**): In a 250 mL two necked round-bottom flask aldehyde (**3**) (1.10 g, 4.0 mmol), 18-crown-6 (104 mg, 0.4 mmol) and benzene (150 mL) were placed. Obtained mixture was purged with argon and brought to reflux, then (1,3-dioxolan-2-ylmethyl)triphenylphosphonium bromide (**8**) (1.72 g, 4.0 mmol) with anhydrous, finely powdered, potassium carbonate (0.36 g, 2.6 mmol) was added in portions every 2 h for 6 h (3x). Afterward the reaction mixture was cooled down and filtered through a pad of silica gel which then was washed using chloroform (250 mL) as an eluent. The solvents from the combined filtrates were removed under reduced pressure and the remaining solid was dissolved in a mixture of tetrahydrofuran (100 mL) and chloroform (30 mL). The concentrated hydrochloric acid (15 mL) was added to the solution and the resulting mixture was vigorously stirred for 45 minutes. The acid was neutralized with concentrated ammonia and after dilution with dichloromethane (100 mL) the organic phase was separated, dried over anhydrous $MgSO_4$ and evaporated to dryness under reduced pressure. The crude product was purified by column chromatography (silica gel, hexanes fraction from naphtha/dichloromethane mixture 1:3 v/v) to give 0.15 g of unreacted substrate **3** and 0.96 g (80% yield) of desired product **4** as yellow solid.
$^{1}$H-NMR (300 MHz, $CDCl_3$) δ (ppm): 9.67 (d, $^{3}J$ = 7.8 Hz, 1H, CHO), 7.69 (d, $^{3}J$ = 15.9 Hz, 1H, $C_{3Vin}$-H), 7.48 (dd, $^{3}J$ = 5.1 Hz, $^{4}J$ = 1.2 Hz, 1H), 7.34 (s, 1H, H-4′), 7.30 (dd, $^{3}J$ = 5.1 Hz, $^{4}J$ = 1.2 Hz, 1H), 7.25 – 7.19 (m, 2H), 7.16 (dd, $^{3}J$ = 5.1 Hz, $^{3}J$ = 3.6 Hz, 1H), 7.05 (dd, $^{3}J$ = 5.1 Hz, $^{3}J$ = 3.6 Hz, 1H), 6.59 (dd, $^{3}J$ = 15.9, $^{3}J$ = 7.8 Hz, 1H, $C_{2Vin}$-H). $^{13}$C-NMR (75 MHz, $CDCl_3$) δ (ppm): 193.86, 144.19, 138.74, 137.38, 135.87, 133.84, 133.58, 129.55, 128.45, 128.37, 128.19, 128.09, 125.76, 124.94, 121.97.

**Prato reaction general procedure:** Aldehyde **3**, **4** or **5** (0.40 mmol), fullerene $C_{60}$ (0.32 g, 0.45 mmol) and sarcosine (45 mg, 0.50 mmol) were dissolved in dry toluene (100 mL) and the resulting mixture was refluxed under argon atmosphere for 24 h. After cooling to room temp., silica gel (10.0 g) was added to the mixture and the solvent was removed under vacuum at 50°C. The remaining was put to the top of the chromatographic column and eluted with toluene – hexane (3:2) to give dark brown amorphous solid.

***N*-methyl-2-([2,2′:5′,2′′]terthiophen-3′-yl)fullero[3,4]pyrrolidine (C$_{60}$TTh):** Aldehyde **3** – 116 mg, 125 mg of product obtained – 30% yield. $^{1}$H-NMR (300 MHz, $CDCl_3$) δ (ppm): 7.72 (s, 1H, H-4′), 7.40 (dd, $^{3}J$ = 5.1 Hz, $^{4}J$ = 1.2 Hz, 1H), 7.23 (dd, $^{3}J$ = 5.1 Hz, $^{4}J$ = 1.2 Hz, 1H), 7.21 – 7.14 (m, 2H + toluene), 7.08 (dd, $^{3}J$ = 5.1 Hz, $^{3}J$ = 3.6 Hz, 1H), 7.02 (dd, $^{3}J$ = 5.1 Hz, $^{3}J$ = 3.6 Hz, 1H), 5.38 (s, 1H. Pyr-$H_A$), 4.94 (d, $^{2}J$ = 9.5 Hz, 1H, Pyr-$H_B$), 4.20 (d, $^{2}J$ = 9.5 Hz, 1H, Pyr-$H_C$), 2.83 (s, 3H, N-$CH_3$). HRMS (ESI) calculated mass for $C_{75}H_{14}NS_3^+$ $[M+H]^+$ : 1024.0283, found: 1024.0281

***N*-methyl-2-(2,5-di(selenophen-2-yl)thiophen-3-yl)fullero[3,4]pyrrolidine** (**C$_{60}$ThSe**)**:** Aldehyde **4** – 148 mg, 115 mg of product obtained – 26% yield. $^{1}$H-NMR (300 MHz, $CDCl_3$) δ (ppm): 8.10 (dd, $^{3}J$ = 5.6 Hz, $^{4}J$ = 1.2 Hz, 1H), 7.90 (dd,

$^3J$ = 5.6 Hz, $^4J$ = 1.2 Hz, 1H), 7.66 (s, 1H, H-4$_{Th}$), 7.43 – 7.20 (m, 3H + $CHCl_3$ + toluene), 5.38 (s, 1H, Pyr-H$_A$), 4.93 (d, $^2J$ = 9.3 Hz, 1H, Pyr-H$_B$), 4.20 (d, $^2J$ = 9.3 Hz, 1H, Pyr-H$_C$), 2.83 (s, 3H, N-$CH_3$). HRMS (ESI): calculated mass for $C_{75}H_{14}NSSe_2^+$ [M+H]$^+$: 1119.9172, found: 1119.9175.

**(*E*)-*N*-methyl-2-(2-[2,2′:5′,2′′]terthiophen-3′-yl-ethenyl)fullero[3,4]pyrrolidine** (**$C_{60}$TThVin**): Aldehyde **5** – 121 mg, 189 mg of product obtained – 45% yield. $^1$H-NMR (300 MHz, $CDCl_3$) δ (ppm): 7.37 (dd, $^3J$ = 5.1 Hz, $^4J$ = 1.4 Hz, 1H), 7.34 (s, 1H, H-4′), 7.32 – 7.22 (m, 2H +$CHCl_3$ + toluene), 7.21 – 7.15 (m, 1H + toluene), 7.15 – 7.07 (m, 2H), 7.02 (dd, $^3J$ = 5.1 Hz, $^3J$ = 3.6 Hz, 1H), 6.65 (dd, $^3J$ = 15.9 Hz, $^3J$ = 9.0 Hz, 1H, H$_{Vin}$), 4.91 (d, $^2J$ = 9.5 Hz, 1H, Pyr-H$_B$), 4.48 (d, $^3J$ = 9.0 Hz, 1H, Pyr-H$_A$), 4.15 (d, $^2J$ = 9.5 Hz, 1H, Pyr-H$_C$), 2.94 (s, 3H, N-$CH_3$) HRMS (ESI): calculated mass for $C_{77}H_{16}NS_3^+$ [M+H]$^+$ : 1050.0439, found: 1050.0446

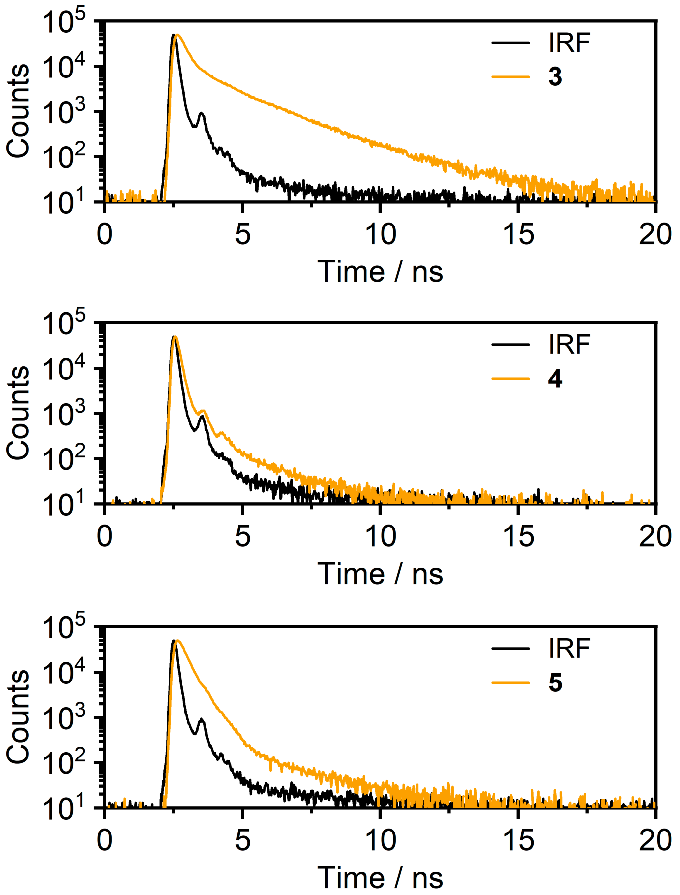


**Figure S1.** TCSPC traces in degassed $CH_2Cl_2$ solutions of **3**, **4**, and **5**, c = $10^{-5}$ M. Collection wavelengths correspond to emission maxima in Figure 2 (middle).
